# Application of CRISPR-Cas System in Human Papillomavirus Detection Using Biosensor Devices and Point-of-Care Technologies

**DOI:** 10.34133/bmef.0114

**Published:** 2025-03-19

**Authors:** Chang He, Yongqi Li, Jinkuan Liu, Zhu Li, Xue Li, Jeong-Woo Choi, Heng Li, Shan Liu, Chen-zhong Li

**Affiliations:** ^1^ Biomedical Engineering, School of Medicine, The Chinese University of Hong Kong (Shenzhen), Shenzhen 518172, China.; ^2^ School of Medicine, University of Electronic Science and Technology of China, Chengdu, Sichuan 610054, China.; ^3^ College of Medical Technology, Chengdu University of Traditional Chinese Medicine, Chengdu 610075, China.; ^4^ Juxintang (Chengdu) Biotechnology Co. Ltd., Chengdu 641400, China.; ^5^ Department of Chemical and Biomolecular Engineering, Sogang University, Seoul 04107, Republic of Korea.; ^6^ Healton Animal Health Biotech Co. Ltd., Neijiang 641000, China.; ^7^ Sichuan Provincial Key Laboratory for Human Disease Gene Study, Department of Medical Genetics, Sichuan Academy of Medical Sciences & Sichuan Provincial People’s Hospital, University of Electronic Science and Technology of China, Chengdu 610072, China.; ^8^ Tianfu Jincheng Laboratory, City of Future Medicine, Chengdu 610072, China.

## Abstract

Human papillomavirus (HPV) is the most common virus for genital tract infections. Cervical cancer ranks as the fourth most prevalent cancer globally, with over 99% of cases in women attributed to HPV infection. This infection continues to pose an ongoing threat to public health. Therefore, the development of rapid, high-throughput, and sensitive HPV detection platforms is important, especially in regions with limited access to advanced medical resources. CRISPR-based biosensors, a promising new method for nucleic acid detection, are now rapidly and widely used in basic and applied research and have received much attention in recent years for HPV diagnosis and treatment. In this review, we discuss the mechanisms and functions of the CRISPR-Cas system, focusing on its applications in HPV diagnostics. The review covers CRISPR technologies such as CRISPR-Cas9, CRISPR-Cas12, and CRISPR-Cas13, along with nucleic acid amplification methods, CRISPR-based signal output systems, and point-of-care testing (POCT) strategies. This comprehensive overview highlights the versatility and potential of CRISPR technologies in HPV detection. We also discuss the numerous CRISPR biosensors developed since the introduction of CRISPR to detect HPV. Finally, we discuss some of the challenges faced in HPV detection by the CRISPR-Cas system.

## Introduction

Human papillomavirus (HPV) is the most common virus causing reproductive tract infections and the most common cause of sexually transmitted diseases in the United States [1,2]. A significant proportion of sexually active individuals, both women and men, are likely to experience an HPV infection or even repeated infections during their lifetime [2]. HPV infection causes no symptoms, and a very small percentage of infections lead to cancer. Ninety percent of HPV infections are eventually cleared from the body, and many of the HPV types contracted do not cause cancer [3]. HPV is a double-stranded DNA (dsDNA) virus that affects both men and women. There are many different types of HPV with more than 200 different genotypes, but the majority of them are not tumor relevant. Noncarcinogenic strains of HPV are responsible for genital warts, which can appear on the labia, vagina, cervix (the opening of the uterus), penis, scrotum, or rectum. The cancerous strains of HPV can cause some different types of tumors, but many people with these HPV strains will never develop any type of HPV-associated cancer [4–7]. HPV is estimated to be responsible for more than 99% of cervical, 90% of anal, 69% of vulvar, 75% of vaginal, 40% of penile, and 70% of opharyngeal cancers [4]. HPV infection in women can result in cervical cancer, which is the fourth most frequently diagnosed cancer among women globally [3]. HPV on the cervix damages the cervical epithelial cells. The body’s immune system removes HPV and heals the damaged cells, and most HPV infections are cleared at this stage. In cases where the body’s immune system proves inadequate in fighting HPV, a progressive accumulation of cellular damage can occur, eventually leading to abnormal changes. Under constant attack by the high-risk HPV (HR-HPV) virus, the damaged cells of the cervix can develop precancerous lesions that progress to break through the basement membrane and cause cancer [8]. Ninety-nine percent of cervical cancers are caused by HPV infection [3]. In rich countries, local policies can get girls to go for HPV vaccination and regular early detection and appropriate treatment. Early screening can detect most precancerous conditions, which are easier to treat. In poor countries, access to these screening initiatives is limited and cervical cancer is often diagnosed when symptoms appear. Moreover, cancer treatments (such as surgery, radiotherapy, and chemotherapy) may be limited. Therefore, cervical cancer has a high mortality rate in these countries. The global mortality rate from cervical cancer (age-standardized rate of 13.3 per 100,000 women in 2020) remains high but could be significantly lowered through targeted interventions implemented at various stages of life [9,10]. Based on data from 65 studies involving 44,769 men, conducted between 1995 January 1 and 2022 June 1, the study estimated the global prevalence of genital HPV infection in men to be 31% [95% confidence interval (CI), 27 to 35] for any HPV and 21% (95% CI, 18 to 24) for HR-HPV. Nearly one-third of men worldwide are infected with at least one genital HPV type, and about one-fifth of men are infected with one or more HR-HPV types. Recent studies show that the prevalence of HPV in males over the age of 15 is immense. It also proves that sexually active men, regardless of age, are a major source of HPV genital infection [11]. Cervical cancer elimination is defined as a country achieving fewer than 4 cases per 100,000 women annually. The World Health Organization (WHO) has implemented various strategies to prevent and control cervical cancer, including vaccination, routine screening, timely treatment, and comprehensive management of invasive cancer [9]. Regular screening is an important part of disease development and control management. Cervical cancer screening includes detection of the HPV virus to detect cancer and precancerous lesions, and then appropriate treatment measures were taken for symptomatic support treatment. The population tested is women without any clinical symptoms. Upon the detection of HPV virus infection or precancerous lesions through screening, prompt and accessible interventions can be administered to prevent or defer the progression to cancer. In the general female population, screening initiation is recommended at the age of 30, with subsequent HPV testing advised at intervals of every 5 to 10 years [10].

**Fig. 1.**
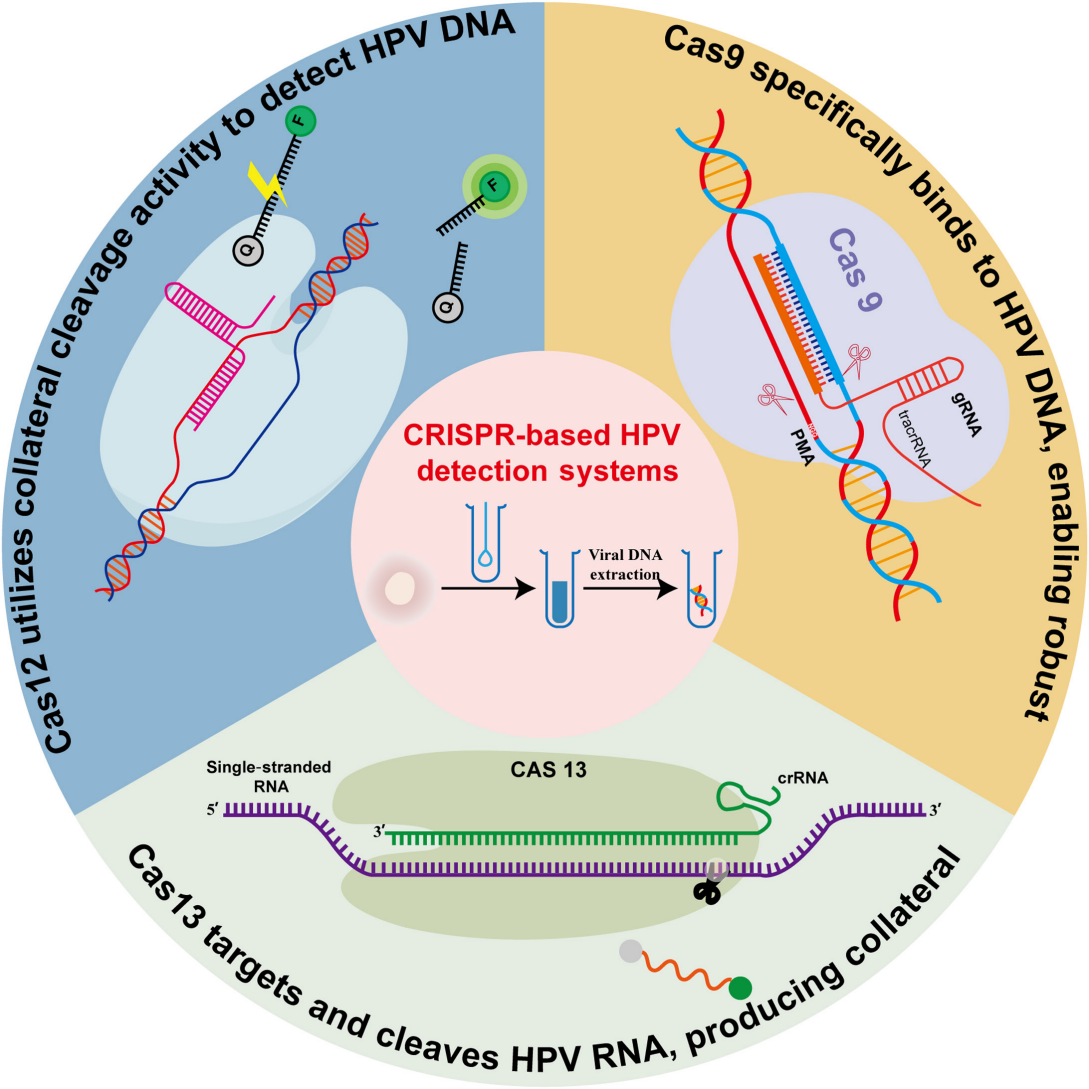
CRISPR-based HPV detection systems utilize Cas9 for specific HPV DNA binding, Cas12 for collateral cleavage of DNA, and Cas13 for targeting and cleaving HPV RNA, enabling robust and versatile diagnostics.

HPV testing is one of the best known and most widely used tests for the detection of HPV in cervical cytology specimens [12–14]. The traditional method of detecting cervical cancer is a cervical cell smear, which is examined under a microscope by an experienced pathologist to determine the presence of abnormal cells. Although cytologic screening has greatly reduced cervical cancer incidence and mortality in many affluent countries, it is a qualitative process with very low sensitivity (as low as 53%). Cytology screening is difficult to implement and sustain in low-income countries and remote areas because it requires highly trained staff and substantial resources [12]. Molecular-level detection methods are widely used for HPV pathogen detection. With the American Cancer Society’s recommendation of HPV detection as one of the guidelines for cervical cancer screening, molecular testing modalities have become particularly important in clinical practice. Since 2001, the Food and Drug Administration (FDA) has approved 5 molecular tests for the detection of HPV in cervical cells [15]. However, the high complexity, long duration, and high cost of these assays have hindered their use in the rapid clinical diagnosis of HPV infection. The large number of commercially available HPV tests on the market makes it difficult to choose the best test for a cervical cancer screening program [16]. Polymerase chain reaction (PCR) stands as the predominant method for nucleic acid detection. Its primary drawback lies in the need for precise temperature cycling, specialized thermal equipment, skilled personnel, a controlled laboratory environment, and a complex multi-step protocol. These requirements preclude swift on-site screening, particularly in remote or underserved areas lacking adequate testing facilities, thereby significantly diminishing the efficiency of HPV virus detection [17]. Therefore, there is an urgent need for a rapid, inexpensive, and highly sensitive point-of-care test for early screening and treatment to prevent the further spread of HPV virus and for early diagnosis and treatment of precancerous lesions. At present, CRISPR-Cas-based nucleic acid detection technology has the following advantages: (a) high specificity, allowing for the detection of single-nucleotide point mutations; (b) high sensitivity, enabling the detection of even single-copy nucleic acids; (c) rapidity, with completion times ranging from 0.5 to 1.0 h; (d) simplicity, allowing on-site execution without the necessity for specialized equipment or personnel; (e) cost-effectiveness, exhibiting a low expenditure for individual tests; (f) ease of development, facilitating the rapid creation of new nucleic acid test kits and similar applications [18]. As shown in Fig. 1, the CRISPR system, as a specific nucleic acid detection platform, has broad application prospects and value in cervical cancer screening.

## Characteristics and Applications of CRISPR

### Brief nomenclature of CRISPR-Cas systems and their features

As a microbial adaptive immune system that possesses endonuclease activity and can specifically resist nucleic acid invasion by phages, transposons, or plasmids, CRISPR is present in about 50% of prokaryotes and about 90% of ancient organisms [18,19]. First discovered in prokaryotes, CRISPR-Cas has powerful gene editing, regulation, and detection capabilities in eukaryote and has been widely used in a variety of basic and applied research [20,21]. The CRISPR-Cas system is composed of CRISPR (clustered regularly interspaced short palindromic repeats) and proteins associated with CRISPR, known as Cas proteins. According to the number of effectors, Cas proteins can be divided into 2 major classes, which are further subdivided into 6 different types depending on their protein usage, and the role of Cas proteins is to cleave base sequences at specific positions [22,23]. Class 1 systems include types I, III, and IV, and class 2 systems include types II, V, and VI.

Class 1 systems use multiple effector proteins to bind to different sites on the guide RNA (gRNA) and DNA nucleic acids and cleave the target DNA, allowing the gRNA to be directed to the target site. In contrast, class 2 systems have only one multidomain protein capable of performing all activities, and class 2 systems include class II, V, and VI, each with unique effector proteins [24–26]. The effector module of class 2 systems consists of a large, multidomain, multifunctional protein with a simple structure and highly efficient gene editing functions and is currently the most widely used in genetic engineering. Due to their simplicity and ease of production, class 2 CRISPR-Cas systems are widely employed in CRISPR diagnostics. Cas proteases in the second class of systems include Cas9, Cas12a/b, Cas13, and Cas14 (Table 1). gRNA is CRISPR RNA (crRNA) during the cleavage process of Cas12a and Cas13 Cas proteases. In contrast, the cleavage process of the 3 proteases Cas9, Cas12b, and Cas14 requires a short piece of trans-activating crRNA (tracrRNA) in addition to crRNA [27,28]. Different Cas proteases have different mechanisms of nucleic acid detection [29]; Cas9 uses target-specific gRNAs to specifically recognize the target sequence. Nucleic acid assays based on Cas12, Cas13, and Cas14 utilize the accessory cleavage activity of Cas proteins to cleave bands when activated [29–31]. When Cas proteins bind to gRNA and target sequences to form effector complexes, their accessory cleavage activity is stimulated, the labeled single-stranded DNA (ssDNA) or single-stranded RNA (ssRNA) reporter genes are cleaved, and the released signals are read by the naked eye or exogenous devices to achieve the detection effect [31].

**Table 1.** Comparison of common CRISPR systems in HPV

Effector	Target type	Guide RNA	Self vs. nonself-discrimination	Cut	Characteristics	Use	Reaction temperature	Detection platform
Cas9	DNA	tracrRNA, crRNA	PAM	Blunt-ended DSB	No collateral cleavage, no secondary structure restrictions	Gene editing, nucleic acid detection	37 °C	CRISPR-Chip
Cas12a	DNA	crRNA	PAM	Sticky-ended DSB	Collateral cleavage, no secondary structure restrictions	Gene editing, nucleic acid detection	37–42 °C	DETECTR
Cas12b	DNA	tracrRNA, crRNA	PAM	Sticky-ended DSB	Collateral cleavage, no secondary structure restrictions	Gene editing, nucleic acid detection	50–60 °C	DETECTR
Cas13	RNA	crRNA	PAM	ssRNA	Collateral cleavage, no secondary structure restrictions	Gene editing, nucleic acid detection	37–42 °C	CARP

### CRISPR-Cas systems for HPV detection

#### CRISPR-Cas12 systems

Type V effector (Cas12, Cpf1) is a class 2 targeting system that has accessory cleavage activity on ssDNA in addition to targeting cis-cleavage of dsDNA, where cis-cleavage is followed by target cleavage to produce sticky ends [32]. Cas12a is the most common member of the Cas12 family. Cas12a, the first member of the Cas12 family to be discovered, has similar institutional components to all enzymes, except that the Cas12a protease requires only a crRNA to guide recognition of the target to activate its activity [33]. Cas12 proteins, including Cas2b and Cas12f (also referred to as Cas14a), rely on both crRNA and tracrRNA for the activation of their enzymatic functions [34,35]. Typically, the protospacer adjacent motif (PAM) of Cas12a, which includes the bacterium Cas12a (LbCas12a) of the family Trichothecaceae and its Acidaminococcus homolog (AsCas12a), is 5′-TTTN, while the PAM of *Bacillus hisashii* Cas12b (BhCas12b) is 5′-ATTN [34]. Cas12 protein binds to crRNA and target DNA to form a complex that activates Cas12 protease activity through RuvC and NUC, 2 cleavage-function elements that cleave the target to form sticky ends. The accessory cleavage activity on nontarget ssDNA occurs in the absence of PAM sequences. This characteristic renders Cas12 promising for signal amplification in nucleic acid diagnostics, thereby prompting the further development of the CRISPR/Cas toolbox for enhanced nucleic acid detection (Table 2) [35,36].

**Table 2.** Applications of nucleic acid detection based on CRISPR-Cas12/Cas9 systems in HPV

CRISPR effector protein	Readout	Target	Preamplification strategy	Assaying time	LOD	Samples	Detection platform	Ref.
Cas12a	Electrochemiluminescence (ECL)	HPV16	Unamplified	100 min	8.86 fM	HPV16 DNA		[70]
Cas12a	Electrochemiluminescence (ECL)	HPV16	Unamplified	20 min	8.3×10^−18^ M	HPV16 DNA heat-inactivated samples	SGGTsensors	[71]
Cas12a	Electrochemiluminescence (ECL)	HPV16	Unamplified		3.2 fM	Clinical sample of cervical cancer patients		[72]
Cas12a	Electrochemiluminescence (ECL)	HPV18	Unamplified			HPV18 DNA		[125]
Cas12a	Photoelectrochemical	HPV16	Unamplified		1.6 pM	HPV16 DNA		[73]
Cas12a	Photoelectrochemical	HPV16	Unamplified		1 pM	HPV16 standards were spiked into human serum specimens		[74]
Cas12a	Photoelectrochemical	HPV16	Unamplified		1.2 pM	Clinical cervical brush samples		[75]
Cas12a	Photoelectrochemical	HPV16/ HPV18/ HPV52	Unamplified	40 min	218 fM	Exogenous HPV-DNA in serum sample		[86]
Cas12a	Photoelectric-signal,paper-based lateral flow assay	HPV16/ HPV18	Unamplified		0.21 pM	HPV16 DNA		[101]
Cas12a	Surface-enhanced Raman scattering	HPV16/ HPV18	Unamplified	20 min	1 pM	HPV16, and HPV18 DNA		[85]
Cas12a	Optical signal	HPV16	Unamplified	70 min	10 fM	Clinical DNA samples	DFM	[126]
Cas12a	Electrochemical	HPV16	Unamplified	50 min	3.22 pM	In bodily liquid (serum samples) by spiking known different concentrations of target HPV16		[76]
Cas12a	Electrochemical	HPV16/ HPV18	RPA	1 h	1 × 10^−18^ M	Clinical human patient samples	M-D3	[90]
Cas12a	Electrochemical	HPV16/ HPV18	LAMP		104 total copies	Plasmid		[77]
Cas12a	Electrochemical	HPV16	Unamplified	60 min	0.48 pM	Target HPV16 DNA		[78]
Cas12a	Electrochemical	HPV16	Unamplified	60 mim	0.2 nM	Artificial mismatched nucleic acid targets (HPV16)		[79]
Cas12a	Electrochemical	HPV16	Unamplified		100 fM	HPV16 target		[80]
Cas12a	Electrochemical	HPV16	RPA		1 pM	Clinical vaginal swab samples		[81]
Cas12a	Electrochemical	HPV16	Unamplified		57.2 fM	Target HPV16 DNA		[127]
Cas12a	Current pulses	HPV16	PCR, LAMP, RPA		3 nM	HPV18 target		[82]
Cas12a	Chemiluminescence	HPV16/ HPV18	HCR/RPA		3 pM	Plasmid/clinical HPV samples	CLE-CRISPR	[83]
Cas12a	Chemiluminescence	HPV16	RPA		17 pM	Clinical HPV samples	CRICED platform	[84]
Cas12a	Fluorescence, lateral flow test	HPV16/ HPV18	RPA	40–60 min		Plasmids	CORDSv2	[123]
Cas12a	Fluorescence, lateral flow test	HPV16/ HPV18	RPA	25 min	16.6 aM	Vaginal or cervical swab samples	CasDOS	[59]
Cas12a	Fluorescence	HPV L1 aptamers	RCA		0.1 ng/ml	Cervical tissue samples, urine samples	FRARI	[92]
Cas12a	Fluorescence	HPV16/HPV18	No amplification	80 min	1 aM	Human clinical samples	CASTI	[57]
Cas12a	Fluorescence, lateral flow test	HPV16/ HPV18	Unamplified	80 min	2.3 fM	HPV18 and HPV16 in serum samples	SPEEDi-CRISPR	[93]
Cas12a	Fluorescence, lateral flow test	HPV16/ HPV18	RPA	30 min	1 × 10^−18^ M	HPV16/18 in patient samples	M3-CRISPR	[94]
Cas12a	Fluorescence	HPV18	Unamplified	30 min	100 aM	Cervical epithelial cells samples for HPV18	PddCas	[58]
Cas13a/12a	Fluorescence	HPV16/ HPV18	RAA	30 min	100 copies per ml	Human cervical epithelial tissue samples	CRISPR-Cas12a/Cas13a dual-channel assay	[95]
Cas13a/12a	Fluorescence	HPV16/ HPV18	LAMP	60 min	10 copy/μl	Human genomic DNA from cell lines	CRISPRD platform	[116]
Cas12a	Fluorescence	19 types of HR-HPV	RPA	40 min	0.26 aM	Patient samples for HPV infection	MiCaR	[99]
Cas12a	Fluorescence, lateral flow test	HPV16/ HPV18	PCR	10 min	0.1 aM	Plasmids/human anal swabs	G-CRISPR	[96]
Cas12a	Fluorescence	13 types of HR-HPV	RPA	35 min	500 copies per reaction	HPV plasmid		[98]
Cas12a	Fluorescence	HPV16/ HPV18	RPA		10 pM	Human anal swabs	DETECTR	[37]
Cas12a	Fluorescence	HPV16/ HPV18	RPA	1 h	100 copies	Purification of HPV DNA	DAMR	[97]
Cas12a	Fluorescence	HPV16/ HPV18/ HPV31/ HPV33/ HPV45/ HPV58/	RPA	20 min	40 copies	HPV DNA and patient samples	CreDiT platform	[128]
Cas12a	Fluorescence	HPV16/ HPV18	RPA	30 min	10^−18^ M	Clinical cervical swab samples	DROPT	[129]
Cas12a	Fluorescence	HPV16/ HPV18/ HPV31/ HPV33/ HPV35/ HPV45/	RPA	45 min	1 copy/μl	Clinical sample		[130]
Cas12a	Fluorescence	HPV16	No amplification		0.1 ng μl^−1^	HPV DNA		[131]
Cas12a	Fluorescence	HPV16/ HPV18	Unamplified			HPV DNA		[132]
Cas12a	Fluorescence	HPV16/ HPV18	RPA	30 min	1 copy/μl	Clinical sample		[133]
Cas12a	Fluorescence	HPV16	Unamplified		3.3 pM	HPV DNA		[134]
Cas12a	Fluorescence	HPV18	Unamplified	30 min	10 fM	HPV DNA		[135]
Cas12a	Fluorescence	HPV16	RPA	70 min	2 pM	Clinical cervical swab samples		[136]
Cas12a	Lateral flow assay	HPV16/ HPV18	RPA		30 copies	HPV DNA	LtCas12a DNA	[102]
Cas12a	Lateral flow test	HPV16	RPA		3.3 aM	Vaginal or urethral discharges	CLIPON	[103]
Cas12a	Lateral flow test	HPV16/ HPV18	RPA	30 min	5 copies per reaction	Clinical samples	TESTOR	[104]
Cas12a	Colorimetry	HPV16	No amplification	70 min	1 pM	HPV16 DNA was spiked in 1% human serum for detection		[109]
Cas12a	Colorimetry	HPV16/ HPV18	Unamplified			Clinical samples		[110]
Cas12b	Fluorescence	HPV16/ HPV18	RPA		1 aM	HPV DNAs in human plasma	CDetection	[39]
Cas9	Optoelectronic	HPV16	RT-PCR			Pseudovirus samples		[111]
Cas9	Fluorescence	HPV16/ HPV18	HRCA	90 min	0.01 ng	Genomic DNA of cervical carcinoma cells	CART	[113]
Cas9	qPCR	HPV16/ HPV18	qPCR	3 h	0.002 ng	Cervical mucus exfoliated cells	CARP	[45]
Cas9	qPCR	HPV16/ HPV18	qPCR		180 copies	Plasmid	ctPCR3.0	[114]
Cas9	qPCR	HPV16/ HPV18	qPCR	3–4 h		Human cervical carcinoma cells	ctPCR	[115]

In 2018, Doudna and her team introduced a CRISPR-Cas diagnostic platform known as DETECTR, which utilizes the Cas12a protein to identify target DNA and subsequently activate its collateral cleavage function [37]. DETECTR is an extremely specific assay capable of accurately identifying HPV16 and HPV18 among various HPV subtypes. The researchers showed that once the Cas12a protein recognizes target DNA, it exhibits nonspecific collateral cleavage activity, degrading surrounding DNA molecules. A biological sample and a DNA probe, which is a single-stranded piece of DNA attached at one end to a fluorescent moiety and at the other end to a bursting agent, are added to the reaction system for Cas12a. When the Cas12-dependent crRNA in the reaction system recognizes and binds to the disease-causing nucleic acid in the biological sample, the side-branching activity that destroys the DNA reporter gene is turned on, and the severed DNA probe releases a fluorophore that is detected by the fluorometer as a stable fluorescent signal. The DETECTR system combines Cas12a with isothermal amplification of target DNA, which can increase the sensitivity of detecting nucleic acids [38]. Teng et al. [39] developed a DNA detection strategy called CDetection, which is mediated by Cas12b and demonstrates greater sensitivity to target sequences than the previously reported Cas12a-based detection platform. The platform is able to discriminate between 2 dsDNA viruses, namely, HPV16 and HPV18.

#### CRISPR-Cas9 systems

The CRISPR-Cas9 system is a class 2 CRISPR system containing RECI, RECII, PAM interaction, HNH, and RucV structural domains, which utilizes Cas9 with tracrRNA and crRNA to identify the target and edit the target gene. Cas9 identifies the PAM and cleaves the target DNA, producing blunt ends. The HNH and RucV domains within Cas9 are responsible for its cleavage activity [31,40,41]. The Cas9 enzyme was one of the first CRISPR-based diagnostic systems with high specificity and the ability to distinguish single-base mismatches (Table 2) [42–44].

In 2018, Zhang et al. [45] developed a Cas9 nuclease-based precaution to detect target DNA and named it CARP. The CARP detection technology is divided into 3 parts: (a) single-guide RNA (sgRNA) is composed of tracrRNA and crRNA and forms a complex with Cas9 to cleave target DNA; (b) ligation of cleaved DNA with DNA ligase; (c) PCR amplification of target DNA. The CARP system enabled the validation of the L1 gene in 2 HR-HPV types, HPV16 and HPV18, and the detection of the E6/E7 genes of 2 HR-HPV types in 2 cervical cancer-positive cervical cancer cell lines. The study performed PCR amplification of the target DNA with high sensitivity and specificity.

Cas9 is a typical targeting dsDNA effector protein in class 2 systems. Unlike other nuclease effectors, Cas9 requires trans-activation of the CRISPR/Cas RNA tracrRNA, which allows the protein to interact with an sgRNA containing sequences complementary to the target region. Cas9 binds to PAM sequences downstream of the target sequence, and cleaved target sequences generate blunt-end double-stranded breaks [46–48]. A homologous gene that is widely characterized by the class 2 V CRISPR/Cas system is Cas12a, which recognizes and cleaves target sequences near the PAM site in dsDNA, just like Cas9. Unlike other systems, Cas12a does not need tracrRNA or additional nucleases for crRNA processing, as it is capable of independently mediating the maturation of its own crRNA. In addition, the Cas12 effector produces sticky ends [48,49].

#### CRISPR-Cas13 systems

The CRISPR-Cas13 system belongs to the class 2 CRISPR systems and is characterized by its RNA-targeting capability. Unlike Cas9 and Cas12, which primarily target dsDNA, Cas13 exclusively targets ssRNA. Cas13 contains 2 essential structural domains: the RNA recognition domain (HEPN domains) and the crRNA-binding domain. Once activated by the target RNA, Cas13 undergoes conformational changes and exhibits collateral cleavage activity, degrading surrounding nontarget RNA molecules in addition to the target RNA [50]. This collateral activity has been harnessed for sensitive and specific nucleic acid detection in CRISPR-based diagnostics [51].

Cas13 does not require tracrRNA like Cas9; instead, it relies solely on crRNA for target recognition. After the crRNA guides Cas13 to the complementary RNA target, the HEPN domains induce catalytic degradation of the target and nearby RNAs [52]. This mechanism has enabled the development of highly sensitive detection platforms such as SHERLOCK (specific high-sensitivity enzymatic reporter unlocking), which couples Cas13-based detection with isothermal amplification techniques to achieve attomolar-level sensitivity [51].

Key advances in Cas13-based systems include its ability to detect RNA viruses, distinguish single-nucleotide polymorphisms (SNPs), and provide real-time fluorescent readouts [25,53]. Additionally, the programmability and versatility of Cas13 make it suitable for applications in infectious disease diagnostics, RNA editing, and transcriptome analysis [54].

## CRISPR-Cas12 for HPV Detection

### Nucleic acid pre-amplification

The main strategy for CRISPR-Cas12a detection is to exploit its nonspecific trans-cutting function by combining it with additional pre-amplification methods to increase the sensitivity [55,56]. The actual detection process has a low concentration of target DNA and requires exponential amplification to generate sufficient target concentration to improve signal output. CRISPR-Cas12a is not sufficient for practical applications when used as a single-signal amplifier and has therefore been combined with different nucleic acid amplifications, such as RPA, RCA, LAMP, PCR, and others [57]. The choice of isothermal amplification method needs to be combined with the reaction conditions of CRISPR-Cas12a, and the compatibility of the 2 assays is an important factor. When the reaction conditions of the amplification reaction and the CRISPR assay are incompatible, the 2 reactions are performed in separate templates. In 2-step assays, the risk of contamination in multiple manual steps of domain amplification needs to be considered [58]. For one-step assays, the balance between the amplification reaction and the CRISPR cutting reaction is worth considering. In the case of RPA amplification, the one-step method leverages Cas12a’s high cis-cleavage activity, which depletes a considerable amount of target DNA during the early phase of the reaction. This depletion facilitates RPA to amplify and accumulate more target DNA, thereby enhancing the detection sensitivity [59].

The main detection strategy of Cas12b is similar to that of Cas12a, but the optimal activity temperature of Cas12b is higher, usually between 50 and 70 °C. Therefore, the Cas12b detection strategy is usually used in detection systems under high-temperature conditions, especially in reaction environments that require higher temperatures, such as specific isothermal amplification methods (such as LAMP). However, the RPA amplification product can be preliminarily detected by other means before inducing the Cas12b side-cutting reaction at a higher temperature [37]. Although the detection strategy of Cas12b is constantly being optimized, especially under high-temperature conditions, it has broad application prospects, but due to its high-temperature requirements, its current application range is still not as wide as Cas12a.

### CRISPR-Cas12 binding to biosensor readout systems

A biosensor is an analytical tool used to detect analytes, integrating a biological element with a physicochemical sensor for measurement. Sensitive biological elements, encompassing tissues, microorganisms, organelles, cell receptors, enzymes, antibodies, nucleic acids, and similar components, are biological or biomimetic materials capable of interacting with, binding to, or recognizing the analyte under investigation. Transducers that convert one signal to another work in a physical manner: optical, piezoelectric, electrochemical, electrochemiluminescence (ECL), etc., generated by the interaction of the analyte with it. The biosensor reader device has an associated electronic device or signal processor that is primarily responsible for displaying the results in a user-friendly manner [60]. Biosensors are currently used in a wide variety of applications, including the detection of pollutants, the detection of disease-causing microorganisms, and the detection of disease biomarkers [61,62]. Each biosensor has its own characteristics, and its operational performance is based on the sensitivity, specificity, repeatability, linearity, and selectivity of the sensor [63,64]. Biosensors have many advantages including accuracy, specificity, sensitivity, sample screening, and rapid results [65]. The CRISPR biosensor system is fast, simple and portable, and highly specific, with specific targeted DNA cleavage properties and nucleic acid accessory cleavage activity, making CRISPR-Cas technology a new technology for genome and molecular editing [16].

CRISPR-Cas12a-based biosensors are a promising new method for nucleic acid detection, which can be used in conjunction with various readout methods (e.g., electrochemical, photochemical, colorimetric, fluorescence, colorimetric, and lateral flow analysis) to detect pathogens and improve the sensitivity of pathogen detection [66,67]. CRISPR-Cas12a-based biosensor systems represent a variety of assay platforms that have been implemented for HPV detection. Many CRISPR-Cas12a assays with different readouts have been developed for the rapid and accurate detection of HPV.

#### E-CRISPR

ECL, a combination of electrochemistry and chemiluminescence, plays an important role in bioanalysis and clinical diagnostics, requiring simple equipment, high sensitivity, low background noise, and a wide range of detectable intervals [68,69]. Combining these advantages, CRISPR-Cas12a-based ECL biosensors have higher sensitivity for HPV detection.

**Fig. 2.**
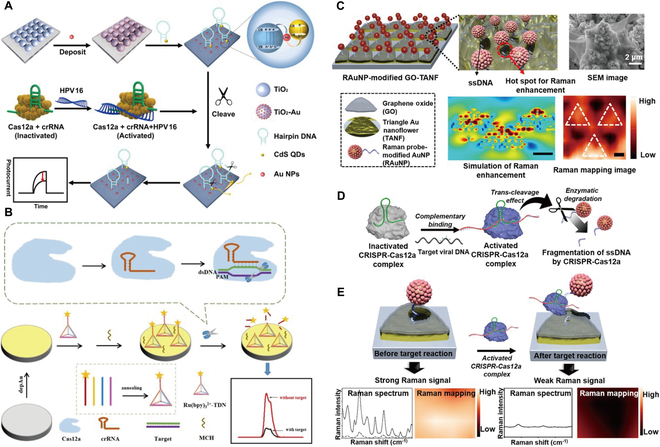
(A) Schematic illustration of CRISPR-Cas12a-based ECL biodetection toward HPV16 [74]. Copyright 2022, American Chemical Society. (B) Schematic illustration of DNA tetrahedron nanostructure and the trans-cleavage activity of CRISPR/Cas12a-mediated ECL biosensor [70]. Copyright 2023, American Chemical Society. Schematic diagram illustrating the strategy to detect viral DNA using SERS-active graphene oxide (GO)/triangle Au nanoflower (GO-TANF). (C) Enhanced SERS signaling. (D) Reverse cleavage effect of CRISPR-Cas12a. (E) ssDNA breakage [85]. Copyright 2021, American Chemical Society.

To date, a variety of ECL biosensors for HPV detection in the CRISPR-Cas12a system have been reported and described [70–84]. In 2022, Li et al. [74] designed a photoelectrochemical biosensor to detect HPV16 via CRISPR-Cas12a-induced disassembly of the Z-scheme heterojunction. Titanium dioxide (TiO_2_)-Au-CdS quantum dots (QDs) Z-scheme system is immobilized in the biosensor. Upon incubation with the target HPV16, the binding of Cas12a-crRNA to the target dsDNA activates the Cas12a protein, and the arbitrary cleavage activity toward the ssDNA leads to the disassembly of the Z-type heterostructures, resulting in a decrease in the photocurrent due to the blocking of electron transfer through the heterojunction (Fig. 2A). The experimental results demonstrated that the Z-scheme sensing system displayed an exceptional photocurrent response to HPV16 across a concentration range of 3.0 pM to 600 nM, with a detection limit as low as 1.0 pM. To improve the sensitivity of HPV16 detection, Yu et al. [70] constructed a sensitive and amplification-free ECL biosensor based on CRISPR/Cas12a and DNA tetrahedral nanostructures (TDNs). The principle is that 3-dimensional TDNs are assembled on the surface of glassy carbon electrodes deposited with Au nanoparticles (AuNPs) to constitute the biosensor. The presence of the HPV16 target activates the accessory cleavage activity of Cas12a-crRNA, which cleaves the ssDNA probe located at the TDN apex, leading to the detachment of Ru(bpy)32+ from the electrode surface and attenuating the ECL signal, thus converting the HPV16 concentration in the system to an ECL signal (Fig. 2B). The TDN-modified sensor reduces the resistance of the CRISPR/Cas12a cut site and improves its cutting performance. The biosensor can complete the detection of HPV16 samples within 100 min with a minimum detection limit of 8.86 fM, which has the advantage of transducing and amplifying the target and improving the efficient Cas12a-crRNA-based trans-cutting activity, thus realizing the sensitive and specific detection of the target samples. In another study, Choi et al. [85] developed a CRISPR-Cas12a-based nucleic acid amplification-free biosensor through a surface-enhanced Raman spectroscopy (SERS)-assisted ultrasensitive detection system. The SERS biosensor system is composed of a graphene oxide (GO)/periodic triangular Au nanoflower (TANF) array with SERS properties, AuNPs functionalized with a Raman probe (RAuNPs), Cas12a complexes, and ambient ssDNA linking the GOTANF and RAuNPs. When the CRISPR-Cas12a complex is activated by the presence of target DNA, it can cleave the ssDNA, causing the Raman probe-functionalized RAuNPs to detach from the sensor, and the weakening of the SERS signal intensity achieves DNA detection (Fig. 2C to E). The CRISPR-based Raman-sensitive system enables the detection of HPV16 and HPV18 without nucleic acid amplification, with high sensitivity and a wide detection range from 1 aM to 100 pM.

On a separate testing platform, Zhan et al. [86] developed a multi-CRISPR-Cas12a-based inductively coupled plasma mass spectrometry (ICP-MS) biosensor. ICP-MS is considered to be an ideal multiplexed and precise tool for the analysis of biological samples, using DNA tetrahedron (DTN) as a support, combined with metal nanoparticles (Au, Ag, platinum, Pd nanoparticles) to greatly amplify ICP-MS signals and further improve the sensitivity and feasibility of the platform for multiplex detection of HPV-DNA (HPV16, HPV18, and HPV52).

Electrochemical biosensors solve the problems of highly skilled operators, high costs, and expensive diagnostics, and with the integration of electrochemical sensors in CRISPR, simple, portable, and sensitive kits have been produced [16,87]. E-CRISPR has demonstrated an accurate, highly sensitive, cost-effective, and commercially available platform for point-of-care testing (POCT) systems in HPV testing.

#### Fluorescent sensors

Fluorescence readout for nucleic acids is a fast sample-reading, very user-friendly method with a wide range of applications and plays an important role in the detection of tumors, viruses, and bacteria [88]. In CRISPR-based nucleic acid detection, the Cas protein is activated in the presence of the target nucleic acid in the reaction system to cleave the ssDNA reporter molecule to release fluorescence [89].

**Fig. 3.**
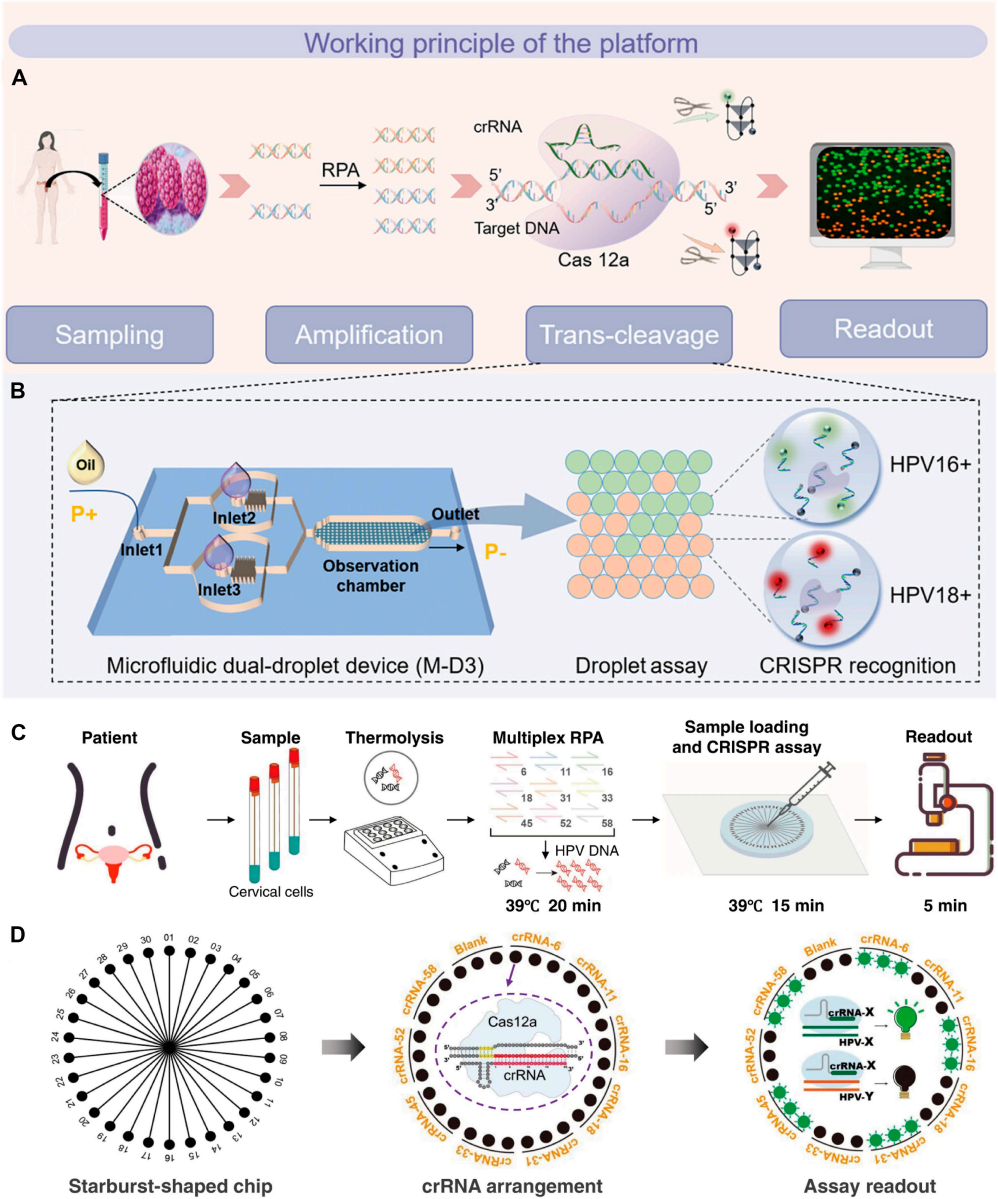
Working principle of the detection platform. (A) Overview of the steps involved in the detection process in M-D3. (B) On-chip assay [90]. Copyright 2023, American Chemical Society. Brief overview of the steps involved in the subtyping process. (C) Sample nucleic acid extraction and amplification. (D) On-chip testing principles [99]. Copyright 2022, Springer Nature.

The complexity of clinical HPV infection determines that one cannot be satisfied with the detection of one type of HPV, and the simultaneous detection of 2 or more HPV types is more applicable in practice, providing more possibilities for experimental research to be translated into products. Zhao et al. [90] developed a simple microfluidic dual-droplet device (M-D3) for the simultaneous detection of HPV16 and HPV18. M-D3 is a photochemical sensor that combines the CRISPR-Cas12a system with RPA amplification [91]. The entire assay process includes sample pretreatment to release nucleic acids, nucleic acid amplification by RPA, CRISPR-Cas12a assay to identify the target transcription reporter and generate a fluorescent signal, as well as performing and obtaining a readout by analyzing images collected on a fluorescence microscope. The M-3 platform encapsulates 2 sets of droplets of deCas12a/crRNA and fluorescent green or red reporter genes, respectively, distributed in parallel: Inlet 1 was pressurized with oil, and inlets 2 and 3 were loaded with RPA amplicons mixed with the CRISPR master solution (containing Cas12a, crRNA, and fluorescently labeled DNA reporters). A vacuum was applied to the outlet, which mainly attracted the reaction solution to the cross section and generated 2 sets of droplets again by the shearing force of the oil. crRNA-HPV16/TBA-TQ and crRNA-HPV18/TBA-TQ were encapsulated in the relevant droplets. When the target is present, Cas12a’s nonspecific cleavage activity is activated, leading to the cleavage of the reporter gene and producing a fluorescent signal for nucleic acid detection. Based on the fluorescence color and intensity, the sample can be distinguished as HPV16/18 positive or negative (Fig. 3A and B). By combining RPA and Cas12a detection, M-D3 can simultaneously detect HPV16 and HPV18 DNA on a chip within 30 min, achieving detection limits of 10 to 18 M (~1 copy/reaction).

Currently, CRISPR-Cas12a-based fluorescent nucleic acid detection platforms are widely used in HPV detection [57,92–97]. In 2018, Doudna’s laboratory developed the DETECTR platform, which for the first time successfully detected HPV16 and HPV18 using the nonspecific ssDNA cleavage function of CRISPR-Cas12a [37]. However, despite these encouraging results, when used for HPV detection, it is time-consuming and labor-intensive because several reactions are required to identify multiple HPV infections. Gong et al. [98] proposed a nongenotyping method to detect 13 HR-HPVs in a single reaction system by combining an isothermal recombinase polymerase amplification (RPA) method with CRISPR-Cas12a technology. In this study, the authors created a primer pool and a crRNA pool capable of detecting 13 types of HR-HPV (HPV16, HPV18, HPV31, HPV33, HPV35, HPV39, HPV45, HPV51, HPV52, HPV56, HPV58, HPV59, and HPV68) in a single reaction. The system consists of an RPA amplification and a Cas12a assay, where the RPA amplification uses a pool from the PGMY/GP 6+ primer set capable of amplifying 13 types of HR-HPV in one reaction and the Cas12a assay uses a crRNA pool. Results can be obtained in less than 35 min using a fluorescence reader. This is the first report using the RPA-Cas12a system to achieve multiple HPV assays in a single reaction and represents a significant advance in the application of the system. The assay is fast (results can be obtained in 35 min) and sensitive (500 copies per reaction). This assay marks a significant step forward in the use of the RPA-Cas12a system and holds substantial promise for overcoming the key challenges currently faced in HPV diagnostics. However, the studies described above were able to perform simultaneous testing for HR-HPV subtypes but could not differentiate between each type, and infection with each HPV type is critical for clinicians to assess the risk of developing cervical cancer. Xu et al.’s [99] workshop proposed a multiplex HPV detection platform called MiCaR, which combines multiplex RPA with CRISPR-Cas12a to achieve fluorescence detection of 9 HPV subtypes targeted by the 9-valent (HPV6, HPV11, HPV16, HPV18, HPV31, HPV33, HPV45, HPV52, and HPV58) HPV vaccines through a microfluidic device. The platform’s assay begins by heating sampled cervical cells to induce the release of HPV DNA. RPA was then performed without DNA extraction to amplify 9 HPV subtypes. The amplification products were loaded onto a microfluidic device, and the amplicons were subsequently tested by a CRISPR-Cas12 a system on the microfluidic device, with reads obtained by an automated fluorescence imaging system. MiCaR detection relies heavily on a starburst-shaped chip (SSChip), which is designed as a network of spokes. Samples are loaded into a central hub, and the spoke microchannels evenly distribute the samples into 30 labeled wells that are preloaded with a specific Cas12a assay mixture containing Cas12a, crRNA, and a fluorescent reporter gene (Fig. 3C and D). When HPV DNA specifically binds to crRNA and activates Cas12a activity, the reporter gene is stolen and a fluorescent signal is released, and the associated wells (3 adjacent wells used as a group to detect the same HPV subtype) will show a bright fluorescent signal. However, if the HPV DNA does not match the crRNA, the background signal will be the same as the blank control (with the reporter gene and Cas12a in the absence of crRNA). This microfluidic “co-amplification, separate detection” strategy enables the simultaneous detection of multiple targets. The use of a single fluorescent probe simplifies system design and data analysis and allows for at-a-glance visualization of results, and the ability to simultaneously test for 3 HPV subtypes ensures accuracy and reliability of results. A series of samples with different plasmid concentrations for 9 HPV subtype (HPV6, HPV11, HPV16, HPV18, HPV31, HPV33, HPV33, HPV45, HPV52, and HPV58) targets were prepared, amplified, and tested in the study, yielding sensitivities of 10–17–10–18 M for all targets. MiCaR was shown to be sensitive in the detection of 100 patient samples at risk for HPV infection and demonstrated strong stability, with on-chip HPV subtyping results highly consistent with clinical results, with a sensitivity of 97.8% and a specificity of 98.1%.

#### Immunochromatographic test strips

With the advantages of simple operation, low technical requirements, rapidity, cost-effectiveness, user-friendliness, and disposability, lateral flow assay (LFA) is now widely used to construct diagnostic methods for nucleic acid testing [100]. In recent years, a variety of biosensor platforms have been constructed by coupling LFA with the CRISPR-Cas12a system [75,93,101–104]. The combination of the LFA platform and CRISPR-Cas12a technology enables direct visualization by the naked eye, facilitating direct result readout and judgment by the assayers, thus eliminating the dependence of conventional fluorescent probe-based CRISPR methods on fluorescent signal reading equipment [105,106]. In biosensing systems, LFA platforms are commonly used to monitor whether Cas nuclease activated by the target undergoes cleavage of reporter molecular probes.

**Fig. 4.**
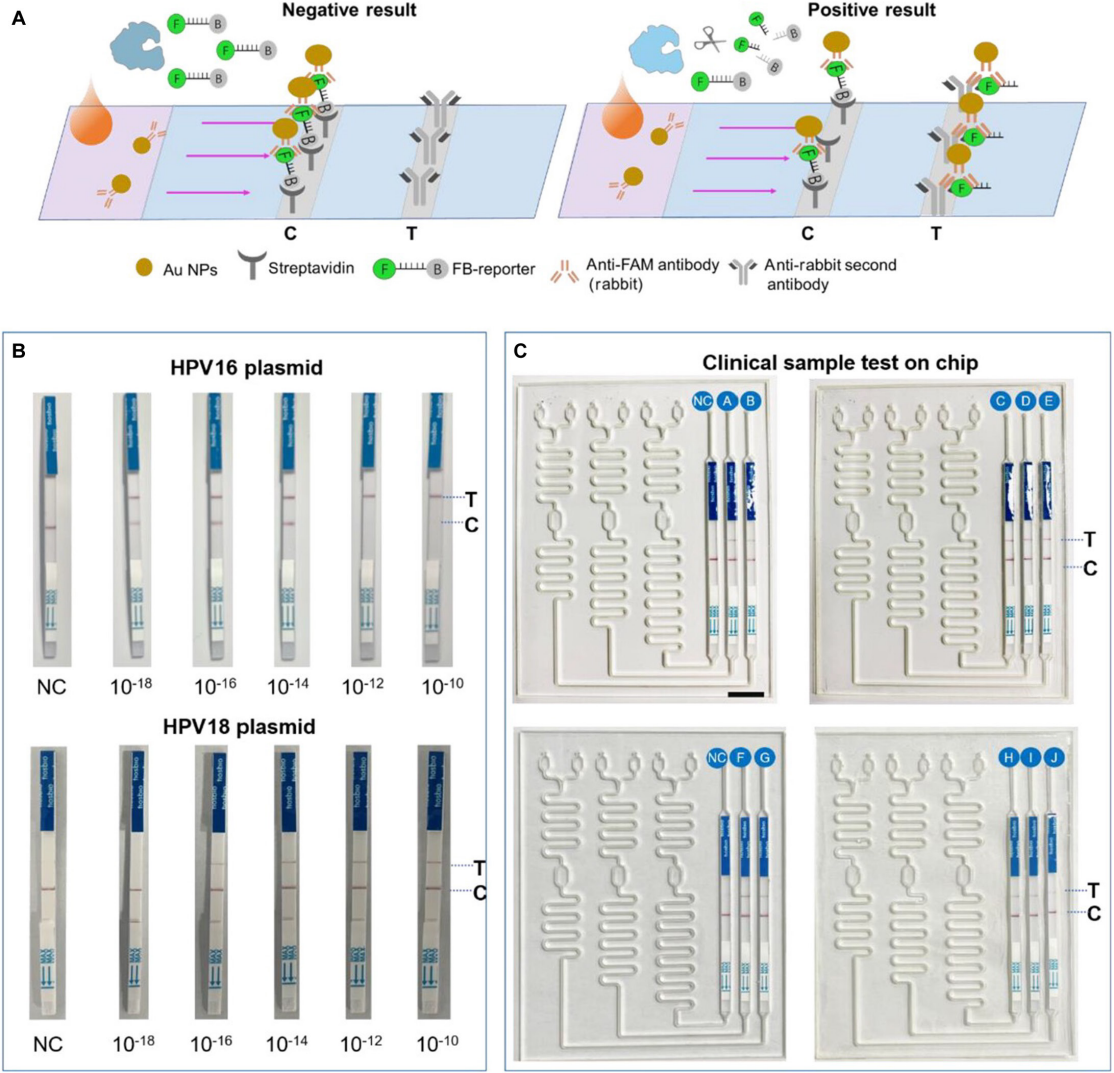
LFD-based detection of HPV16 and HPV18 plasmids and in patient samples. (A) Scheme showing the mechanism of the LFD-based readout. (B) Testing of the amplified HPV16/18 plasmids with LFDs. (C) On-chip testing of HPV16 and HPV18 in clinical samples based on the LFD readout [94]. Copyright 2023, American Chemical Society.

Zhou et al. [94] reported a multiplexed microfluidic platform called M3-CRISPR that combines RPA and CRISPR technology for the detection of multiple HPV subtypes. An optimized lateral flow dipstick (LFD) was introduced into the M3-CRISPR system to enable visual detection of HPV DNA. The reaction principle of the LFD is shown below. As shown in Fig. 4, the test strip is encapsulated with 2 lines on the nitrocellulose membrane, the control line on the left, encapsulated with streptavidin (SA), and the test line on the right, encapsulated with anti-FAM antibody. In negative samples, the reporter gene (with FAM and biotin markers) binds to the anti-FAM antibody/AuNP affix at the binding pad to form a complex and is captured by the SA encapsulated on the control line. In positive samples, part of the reporter gene is cleaved into fragments by the Cas enzyme, and the intact reporter gene in the complex can still be captured by SA to show the control line. The broken reporter gene in the complex (unlinked biotin) flows through the control line and binds to the anti-rabbit secondary antibody to show the detection line. The team tested a series of RPA-amplified HPV16 plasmids and HPV18 plasmids with test strips, and all samples except the negative control showed color on the detection line, resulting in a detection sensitivity of 10^−18^ M for HPV16 and 10^−16^ M for HPV18. M3-CRISPR evaluated 2 negative clinical samples and 10 samples positive for either HPV16 or HPV18 to detect HPV in clinical specimens using LFD-based readouts. The assay successfully identified all samples, demonstrating its effectiveness. M3-CRISPR offers a reliable, portable, and low-cost method for HPV infection detection.

#### Colorimetry

Chromaticity sensors allow for direct visualization of results and are easy to move around and are widely used to identify targets with the naked eye [107]. Colorimetric sensors enable the CRISPR-Cas system to be a simple colorimetric nucleic acid detection system that produces visible color signals without the need for heavy instrumentation, thereby reducing the cost of detection and increasing the number of diseases detected [16,108].

**Fig. 5.**
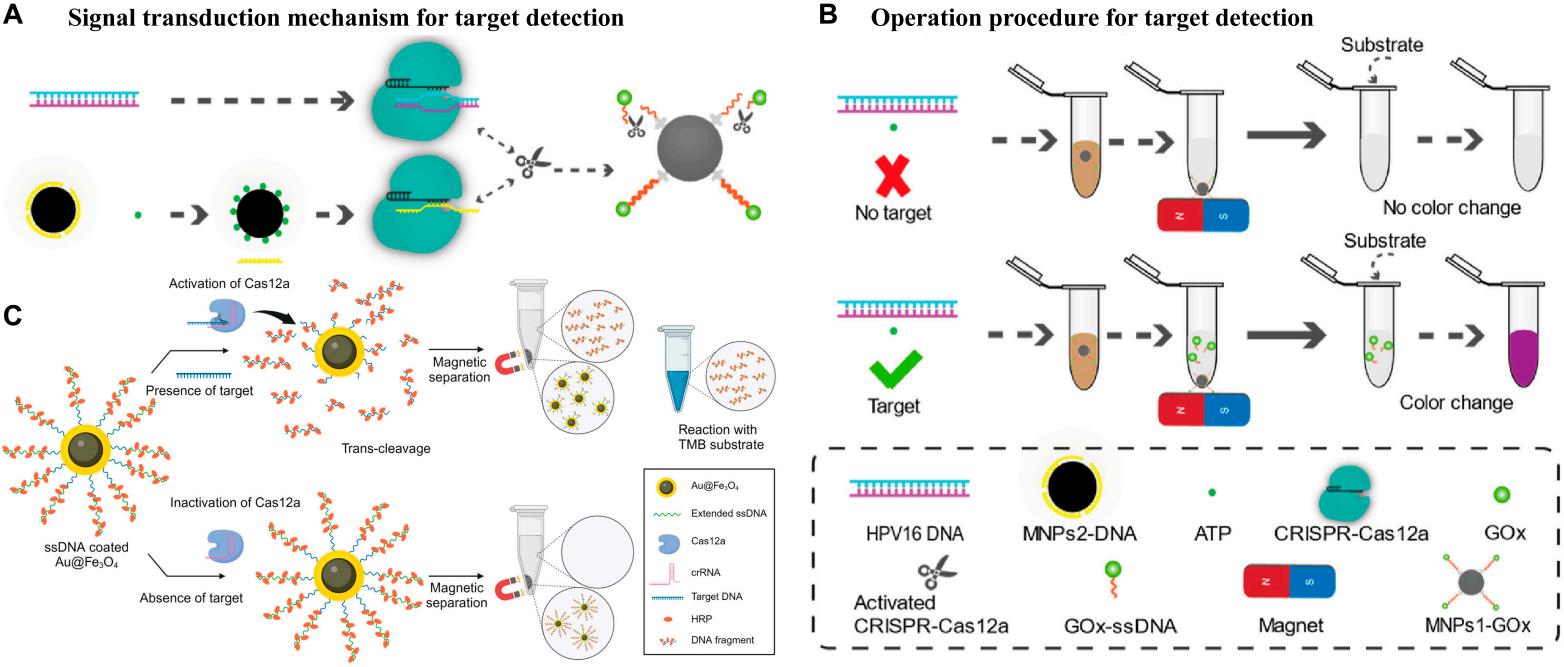
(A and B) Schematic diagram of CRISPR-Cas12a colorimetric detection of HPV16 [104]. Copyright 2024, Elsevier. (C) Schematic of a CRISPR-based DNA biosensor for high-sensitivity colorimetric viral DNA detection using magnetic enzyme complexes [109]. Copyright 2024, MDPI.

Gong et al. [109] recently developed a 2-enzyme cascade amplification strategy based on CRISPR-Cas12a and glucose oxidase (GOx) for the detection of HPV16, which employs a colorimetric readout mode. The reaction principle of this method is that when HPV16 is present in the reaction system as a nucleic acid target, Cas12a is activated as an ssDNA enzyme to cleave the ssDNA [ssDNA with magnetic nanoparticles 1 (MNPs1) and GOx attached at both ends], causing GOx to be released from the surface of the MNPs1 into the supernatant, which then catalyzes the production of a visible purple color by the colorless substrate (Fig. 5A and B). When HPV16 DNA is absent, the MNPs1-GOx complexes remain intact and can be separated using magnetic extraction. The assay’s detection limit for HPV16 DNA is as low as 1 pM, and the resulting colorimetric signal from the target analyte is easily visible to the naked eye. In addition, Park et al. [110] developed a CRISPR-Cas12a-based viral DNA detection system for the detection of HPV, utilizing multi-enzyme-modified Au@Fe_3_O_4_ nanoparticles to enhance signal amplification. This method eliminates the need for traditional nucleic acid amplification by using elongated ssDNA with multi-enzyme modifications to improve detection sensitivity (Fig. 5C). Specifically, when HPV16 DNA is present, Cas12a is activated to cleave the ssDNA attached to the multi-enzyme complex, releasing horseradish peroxidase (HRP) into the supernatant. In the presence of hydrogen peroxide, these HRPs catalyze the substrate to produce a visible blue color, indicating the presence of HPV16 DNA. The system achieved a detection limit of 0.25 nM, and the color change is easily observable without the need for complex equipment, making the method suitable for POCT.

## CRISPR-Cas9 for HPV Detection

**Fig. 6.**
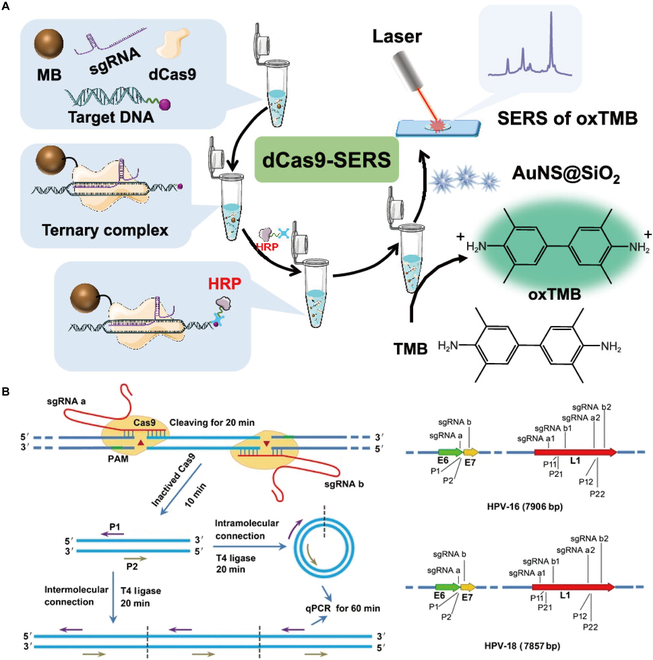
(A) Detection schematic diagram of the method based on CRISPR/dCas9-assisted HRP-catalyzed signal amplification [110]. Copyright 2023, American Chemical Society. (B) Schematic diagram of the CRISPR/Cas9 workflow and detection of genes for HPV16 and HPV18 as proof of principle [113]. Copyright 2018, Springer Nature.

Cas9-based nucleic acid detection relies on the ability to specifically recognize target sequences and specific gRNAs designed for target detection (Fig. 6A) [29,111,112]. In 2018, Zhang and co-workers [111,113] developed a new method called CARP, which is based on CRISPR-Cas9 and combined with PCR methods to achieve detection of HPV16 and HPV18 (Fig. 6B). Based on the reaction principle of Cas9, 2 methods, ctPCR and ctPCR3.0, were developed and applied for the detection of HPV16 and HPV18 [114,115]. The ctPCR method was validated by detecting the L1 and E6-E7 genes of 2 HR-HPV types (HPV16 and HPV18) in cervical cancer cells and clinical specimens. ctPCR 3.0 detects target DNA in a single homogenization step and does not require the tubes to be opened in the middle of the process, reducing the assay time to 2 h. The technology was validated by cloning and testing 10 high-risk types of HPV L1 genes. The method also successfully identified the L1 and E6-E7 genes of 2 HR-HPV types, HPV16 and HPV18, within the genomic DNA of HeLa and SiHa, 2 HPV-positive cervical cancer cell lines. By changing the nucleic acid amplification method, Zhang et al. [113] developed a CRISPR-associated HRCA technique (CART) method by combining the CRISPR-Cas9 system and the rolling circle amplification (RCA) amplification method for rapid and sensitive detection and typing of target DNA113. The entire detection process of the CART method is completed in less than 90 min, and a small amount of substrate DNA can be detected to achieve the lowest detection line for 0.01 ng, and has detected HPV types of HPV16, HPV18, HPV6, HPV11, HPV33, HPV35, HPV40, HPV45, and HPV51.

## CRISPR-Cas13 for HPV Detection

**Fig. 7.**
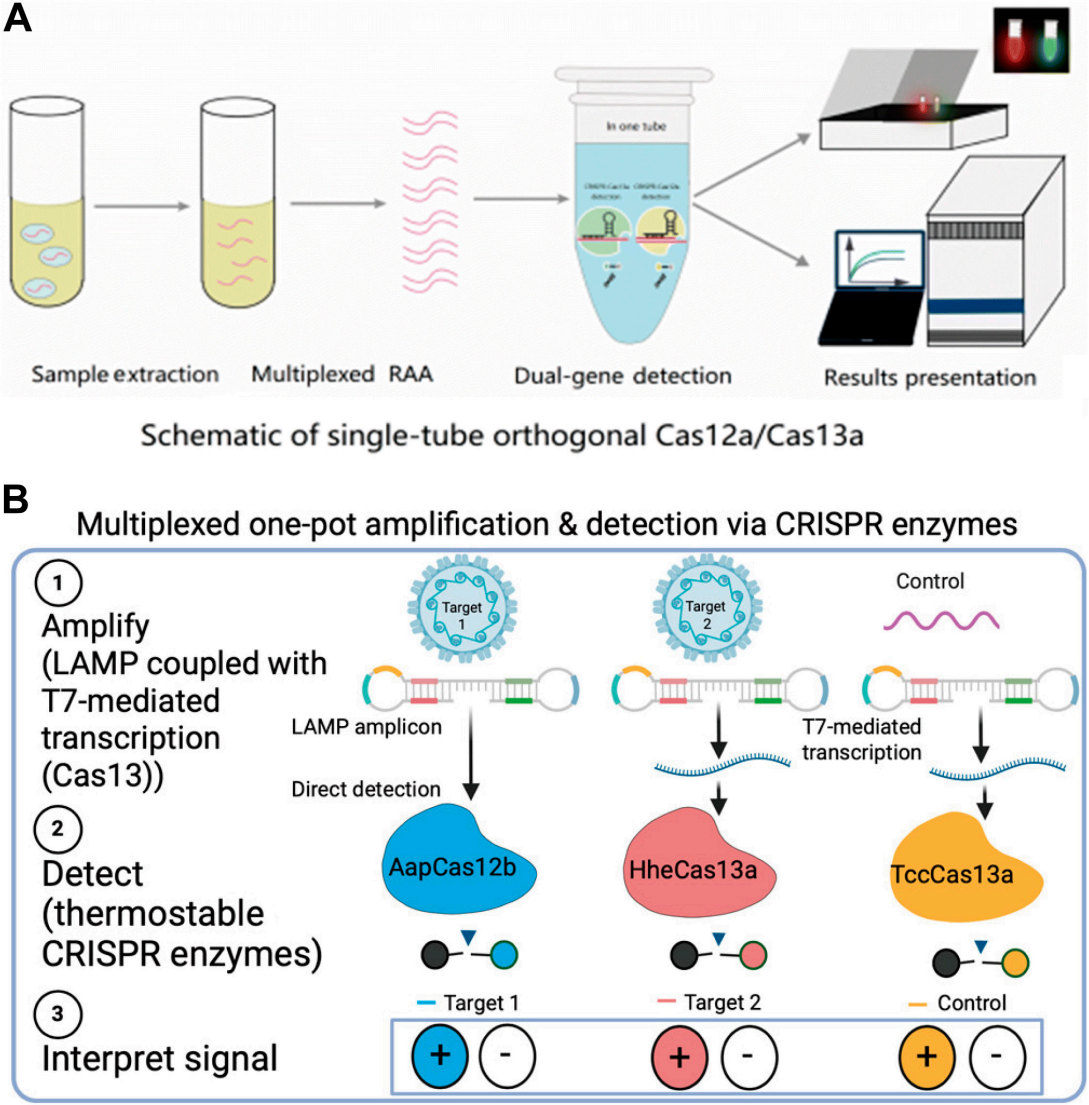
(A) Orthogonal Cas12a/Cas13a detection enables dual-color fluorescence visualization of HPV16/18 through target-specific cleavage of DNA and RNA reporters [95]. Copyright 2022, RSC Pub. (B) Multiplexed one-pot CRISPR detection integrates amplification, thermostable CRISPR enzymes, and signal interpretation for HPV diagnostics [116]. Copyright 2024, American Chemical Society.

Recent advances in CRISPR-Cas13-based diagnostics have greatly improved the sensitivity and versatility of pathogen detection systems, offering new opportunities for multiplexed and accurate detection (Fig. 7). It is worth noting that Zheng et al. [95] introduced a dual-channel CRISPR-Cas13a/Cas12a system for rapid and highly sensitive detection of HPV16/18. The key innovation includes combining multiple recombinase-assisted amplification (RAA) to simultaneously detect and type HPV16 and HPV18 in a single reaction tube. Ghouneimy et al. [116] introduced a multiplex CRISPR-Cas12b/Cas13a detection system, termed CRISPRD, for the rapid and highly sensitive identification of HPV16 and HPV18. The platform combines multiple layers of isothermal amplification with orthogonal CRISPR effectors, enabling the simultaneous detection of HPV16, HPV18, and an internal control (RNase P) in a single reaction tube with a detection limit of 10 copies. In addition, their work provides another idea that the combination of Cas12 and Cas13 may improve the accuracy of detection.

## Application of POCT in HPV Detection

The availability of quick and easy-to-use diagnostic kits is crucial for the effectiveness of any healthcare system. POCT is a medical test that refers to a fast and convenient clinical test performed at the patient’s side. POCT technology allows for rapid test results without the need for specialized technical operators [31,117,118]. POCT devices are often required to meet reliable standards (affordable, sensitive, specific, user-friendly, fast and reliable, no equipment required, deliverable to the end-user) in order to provide rapid and selective patient information in the field for personalized care or to provide a rapid prediagnosis of an infection, often bypassing traditional time-consuming methods and the need for specialized laboratories [119]. The aim of POCT is to deliver real-time, practical information about a patient’s condition to clinicians, enabling more informed therapeutic decisions. This approach helps improve patient outcomes by reducing critical conditions, morbidity, and mortality. Bedside testing can be performed in a variety of settings, including hospitals, homes, or other locations. Depending on the format, bedside devices can be categorized as “removable”, “portable,” or “handheld” [120]. Biosensors are the foundation of POCT diagnostic testing due to their rapid signal readout, economical transduction elements, and manageable sensing platforms [121]. The combination of CRISPR-based molecular assays with the latest sensor technologies will revolutionize the POCT molecular diagnostic market with the development of molecular assays that are more sensitive, more specific, easier to use, higher throughput, faster, smarter, and more efficient [16,122].

CRISPR-based biosensors play an important role in HPV detection and are committed to applied research in POCT. To enhance its potential for POCT, Lin et al. [123] developed a compact and portable detection platform called hippo-CORDS (heating and imaging powerfully portable device for one-pot Cas12a-based onsite and rapid detection system), specifically designed for the detection of HPV16 and HPV18. The integration of one-step pipetting with a low-cost device made it an ideal option for POCT. Tian et al. [93] developed a POCT nucleic acid assay called solid-phase extraction and enhanced detection assay integrated by CRISPR-Cas12a (SPEEDi-CRISPR). The study demonstrated that the SPEEDi-CRISPR assay provides improved sensitivity for the detection of HPV18 with a limit of detection (LOD) of 2.3 fM and excellent specificity for differentiating HPV18 from HPV16. Fluorescence signals generated by SPEEDi-CRISPR can be quantified using benchtop instruments like a qPCR machine or plate reader, or alternatively paired with a smartphone-based portable device or LFA for visual readout, catering to the requirements of POCT. Xue et al. [58] developed a polydisperse digital droplet CRISPR-Cas-based assay (PddCas) for the rapid and ultrasensitive amplification-free detection of viral DNA/RNA with minimal instruments. The study used the PddCas to successfully detect HPV18 in clinical samples. Tang et al. [103] introduced a novel approach named CLIPON (CRISPR and large DNA assembly induced pregnancy strips for signal on detection). Their study showcased the versatility of CLIPON by achieving sensitive and specific detection of HPV genomic DNA, making it a promising tool for biosensing and POCT diagnostics.

## Conclusion

The CRISPR/Cas system has demonstrated tremendous capabilities in the nucleic acid diagnosis of HPV. Based on the powerful and highly specific features of the CRISPR/Cas system, ultra-sensitive, low-cost, and rapid nonlaboratory test kits for HPV detection will be developed. This will greatly improve existing diagnostic tools and could enable early and widespread screening for HPV infection in areas or countries with limited healthcare resources, thereby reducing the incidence of cervical cancer and slowing the progression of the disease [124]. Various platforms based on the CRISPR-associated nuclease Cas9 and Cas12a for the detection of HPV with a focus on Cas12a are outlined in this review. Good progress has been made in enabling POCT diagnosis with the development of the Cas12a system.

However, CRISPR-based HPV nucleic acid testing is still in the early stages of development, with almost all research confined to the laboratory, and many obstacles that may prevent rapid translation of laboratory results into practice. Potential challenges include the following: (a) Specimen collection challenges: Collection of HPV specimens from cervical/vaginal exfoliated cells in women and exfoliated cells from various anatomical sites in men poses difficulties and is reliant on clinician expertise. Ideal specimens, such as menstrual blood in women and urine in men, offer greater accessibility for target detection. (b) Focus on high-risk types and detection accuracy: Current HPV testing predominantly targets high-risk types with significant carcinogenic potential. Given the diversity of HPV types, achieving sensitive and accurate detection at the single-molecule level for a range of types with high sequence homology is crucial. For example, the combination of CRISPR-Cas12 and Cas13 for HPV detection offers advantages such as dual-target detection, improved specificity, enhanced sensitivity, multiplex analysis, faster detection speed, and greater robustness. By simultaneously detecting HPV DNA and RNA, it provides broader viral information, reduces false positives, and maintains high sensitivity in low-concentration samples. (c) Stringent experimental conditions and reagent storage: Existing experimental conditions for HPV testing demand meticulous requirements, including the preservation of reagents like Cas protease and gRNA at low temperatures. Addressing the challenge of storing, transporting, and utilizing nucleic acid reagents at room temperature is an urgent issue. (d) Complexity of clinical infections and multiplex detection: Clinical HPV infections exhibit complexity and variability. Current laboratory testing primarily focuses on 1 or 2 HPV detections, and multiple simultaneous detections do not ascertain the HPV type. Obtaining clinical specimens poses challenges, and developing methods for micro-sample multiplex HPV detection with type identification is clinically imperative. Many existing CRISPR diagnostic tests lack solutions for multi-detection and quantitative detection. (e) Optimization and simplification of CRISPR diagnostic procedures: Optimizing and simplifying CRISPR diagnostic procedures is pivotal for transitioning from laboratory to clinical practice. While many HPV tests incorporate nucleic acid amplification before CRISPR reaction to enhance sensitivity, eliminating this step without compromising diagnostic system sensitivity is crucial. This not only saves time but also reduces the risk of amplification bias and sample cross-contamination, especially considering the susceptibility of isothermal amplification methods to aerosol contamination and the associated potential for false-positive interpretations.

In summary, the adoption of CRISPR-based methodologies holds the promise of addressing the increasing need for swift and precise HPV diagnostic tests, particularly in resource-constrained environments. This paves the way for the potential development of cost-effective, rapid, sensitive, accurate, and suitable HPV bedside diagnostics, offering promising prospects for widespread implementation on a large scale.
